# Migrants in Swedish sexual and reproductive health and rights related policies: a critical discourse analysis

**DOI:** 10.1186/s12939-022-01727-z

**Published:** 2022-09-05

**Authors:** Nada Amroussia, Charlotta Holmström, Pernilla Ouis

**Affiliations:** 1 Centre for Sexology and Sexuality Studies (CSS), Faculty of Health and Society (HS), Nordenskiöldsgatan 1, 211 19 Malmö, Sweden; 2 Department of Women’s and Children’s health, Uppsala University, Uppsala, Sweden; 3 School of Health and Welfare, Halmstad University, Halmstad, Sweden

**Keywords:** SRHR, Migrants, Critical discourse analysis, Policy analysis, Sweden

## Abstract

**Background:**

Previous research has shown that migrants in Sweden are disadvantaged in terms of sexual and reproductive health and rights (SRHR). SRHR policies might play a crucial role in shaping migrants’ SRHR outcomes. The purpose of the study was to critically examine: a) how migrants were represented in the discourses embedded within Swedish SRHR-related policies, and b) how migrants’ SRHR-related issues were framed and addressed within these discourses.

**Methods:**

Critical discourse analysis (CDA) was used to analyze a total of 54 policy documents. Following Jäger’s approach to CDA, discourse strands and entanglements between different discourse strands were examined.

**Results:**

Our findings consisted of three discourse strands: 1) “Emphasizing vulnerability”, 2) “Constructing otherness”, and 3) “Prioritizing the structural level or the individual level?”.

Migrants’ representation in Swedish SRHR-related policies is often associated with the concept of vulnerability, a concept that can hold negative connotations such as reinforcing social control, stigma, and disempowerment. Alongside the discourse of vulnerability, the discourse of otherness appears when framing migrants’ SRHR in relation to what is defined as honor-related violence and oppression. Furthermore, migrant SRHR issues are occasionally conceptualized as structural issues, as suggested by the human rights-based approach embraced by Swedish SRHR-related policies. Relevant structural factors, namely migration laws and regulations, are omitted when addressing, for example, human trafficking and HIV/AIDS.

**Conclusions:**

We conclude that the dominant discourses favor depictions of migrants as vulnerable and as the Other. Moreover, despite the prevailing human rights-based discourse, structural factors are not always considered when framing and addressing migrants’ SRHR issues. This paper calls for a critical analysis of the concept of vulnerability in relation to migrants’ SRHR. It also highlights the importance of avoiding othering and paying attention to the structural factors when addressing migrants’ SRHR.

**Supplementary Information:**

The online version contains supplementary material available at 10.1186/s12939-022-01727-z.

## Background

Migration is an important component of the social, political, and economic reality of our globalized world. Although migrants are particularly vulnerable to poor sexual and reproductive health (SRH) outcomes such as sexual violence, sexually transmitted infections (STIs), and pregnancy complications, their sexual and reproductive health and rights (SRHR) have often not been fully addressed [1, 2]. In Sweden, SRHR have been prioritized in the national and international policies. Despite the official commitment to SRHR, previous literature has pointed out that migrants^1^ in Sweden are disadvantaged in terms of SRHR outcomes and access to SRH services. For instance, studies have shown that foreign-born women are at higher risk of exposure to interpersonal violence [3], and have an increased risk of mortality due to interpersonal violence compared to Swedish-born women [4]. Foreign-born women are also less prone to benefit from planned routine antenatal care compared to Swedish-born women [5]; and migrant women have a higher risk of adverse pregnancy outcomes, such as gestational diabetes and stillbirth [6]. Migrant women are also less likely to use contraception than non-migrant women [7], and can lack knowledge about where to turn for contraceptive counseling and HIV testing [8].

Previous literature focusing on migrant youth has also pointed to multiple barriers faced by this group when accessing SRH services and information [9, 10]. These barriers include language, and financial barriers, in addition to the lack of knowledge about the Swedish health system [10]. Norms related to sexuality might also represent a barrier to accessing SRHR-related information for young migrant women [9]. A study conducted in the region of Uppsala among a sample of 1063 ninth-grade students revealed that 66% of girls and 35% of boys having both parents born outside of the Nordic countries were expected not to have sex before marriage [11].

Migrants’ SRHR are influenced by a large array of factors operating at different levels, including the individual and structural levels. At the individual level, factors such as the level of social support, familiarity with the healthcare system, and language proficiency might influence migrants’ SRHR [12]; whereas, at the structural level, the legal and policy frameworks play a crucial role in shaping migrants’ SRHR [12–16] along with factors such as migration status and socio-economic status [12]. These legal and policy frameworks include what Villalonga-Olives et al. [14] defined as “migration regime” i.e., the system of laws, regulations, policies, and institutions in the host country. Drawing on the results of a systematic review focusing on birth and pregnancy outcomes among immigrant women in the United States and Europe, the authors [14] argue that the “migration regime” represents “the main driver” of migrant health i.e., the main important factor influencing migrant health outcomes. The legal and policy frameworks also include integration policies and policies related to the provision of SRH services in the host country [12, 13, 15].

SRHR policies might be an important component of these legal and policy frameworks, and understanding how migrants’ SRHR are addressed in these policies might provide important insights into possible explanations of migrants’ SRHR outcomes. Despite its importance, a paucity of research has examined SRHR policies in relation to migration [13, 17]. By focusing on the Swedish context, this study seeks to generate a comprehensive analysis of Swedish SRHR-related policies as it is related to migration. Policies might not only seek to address social problems, but can also contribute to shaping the representations of specific groups in society [18]. The purpose of the study was to critically examine: a) how migrants were represented in the discourses embedded within Swedish SRHR-related policies, and b) how migrants’ SRHR-related issues were framed and addressed within these discourses.

It is worth noting that there is “no universally accepted definition for migrants” [19, 20]. In this study, migrants are understood as foreign-born people who have migrated and settled in Sweden for different reasons (political, economic, humanitarian…) regardless of their legal status and length of stay. While the primary focus of the study was on migrants, people with a foreign background were also included in the analysis.^2^ This term is commonly used in the Swedish context to refer to a larger group, including foreign-born residents and Swedish-born residents with two foreign-born parents.

## Methods

### Global and Swedish context

This policy analysis focused on SRHR-related policy documents published after the mid-90 s until 2020 in the context of shifting migration policies in Sweden and a shifting discourse on SRHR at the global and national levels.

#### Migration policies in Sweden

Compared with other countries in the European Union (EU), Sweden was considered to have an open migration policy and a generous asylum policy that “went beyond the EU minimum standards” [21]. From 1998 to 2014, asylum was granted to bigger groups than in other EU countries and permanent residence permits were given to people given international protection. Since the mid-70 s, the Swedish migration policies were generally framed within the multicultural framework. In 1975, the immigrant and minority policy recognized the right to be different, in a marked departure from the previous assimilation approach. In 1997, Sweden adopted a new integration policy, called “Sweden, the Future and Diversity-from Migration Policy to Integration Policy” [22]. This policy and its effects were interpreted differently. While some scholars argue that this policy represented an official retreat from multiculturalism in Sweden [23, 24], other scholars argue that Sweden is one of the European countries where the multicultural policies were strengthened in the period between 1980 and 2010 [25].

Sweden was particularly affected by the “refugee crisis” in 2015. According to the Organization for Economic Cooperation and Development (OECD) [26], Sweden has received over 163 000 asylum seekers in 2015 representing “the largest per capita inflow ever recorded in an OECD country”. In response to this situation, external borders controls were introduced, and restrictive asylum measures were adopted [21]. These measures consisted in granting only temporary residence permits for people receiving asylum and restricting family reunification [27].

#### Global and Swedish recognition of SRHR

The mid-90 s were marked by a breakthrough in international policies regarding SRHR. In 1994, reproductive rights were globally recognized as human rights at the International Conference on Population and Development (ICPD) conference [28]. The ICPD program of Action was considered “the first global agreement that created a common language” for conceptualizing and defining SRHR [29]. The global recognition of reproductive rights was reinforced in 1995 in the Beijing Declaration and Platform of Action [29, 30]. The Guttmacher-Lancet Commission [29] developed an integrated definition of SRHR. Within this definition, SRH is defined as a state of physical, emotional, mental, and social wellbeing in relation to all aspects of sexuality and reproduction. Sexual and reproductive rights imply having the right to make decisions governing one’s own body and having access to services supporting that right. Sexual and reproductive rights include, among others, the right to have one’s own bodily integrity respected, the right to freely define one’s own sexuality, and the right to decide whether, when, and whom to marry [29].

Sweden has embraced the concept of SRHR that was incorporated in different policies related to, among others, maternal health, HIV/AIDS, and violence against women, including female genital mutilation (FGM). Gender-based violence, including violence against women, represents a breach of a fundamental sexual and reproductive right. i.e., the right to have one’s bodily integrity, privacy, and personal autonomy respected.

These policies were defined as Swedish SRHR-related policies in the current study. It is worth noting that the Swedish national SRHR strategy was published only in October 2020 [31], 14 years after adopting an international policy on SRHR [32]. This international policy was not considered in the current study as it focuses only on Sweden’s international work and collaboration [32].

### Methodological approach

In this study, Critical discourse analysis (CDA) [33–36] was used to explore how migrants’ SRHR were represented in Swedish SRHR-related policies. A special attention was paid to the representation of migrants and people with a foreign background in the policy discourses.

While there are different approaches to CDA, all these approaches concur that CDA is a problem-oriented methodology that does not focus on the linguistic items per se but rather on social issues related to power abuse and social inequalities [34–36] or what Fairclough (2013) called “social wrongs”. Fairclough [34] defined CDA as a systematic exploration of the relationships “between discursive practices and wider social and cultural structures, relations and processes”. CDA is also defined as an investigation of how discourses are shaped by the relations of power [34] and an approach that focuses on the role of discourse in “the (re)production and challenge of dominance” [35].

Within the CDA approach, discourse is understood as “socially constituted” i.e. shaped by a wider social context, and socially constitutive i.e. it frames identities, shared attitudes, and knowledge [34]. Critical discourse analysts placed emphasis on the social representation of the “Other” i.e. the non-dominant groups enacted by the discourse [35]. This representation can be shaped by dominance relations and it can justify maintaining and reproducing these relations [35].

CDA has been increasingly used for analyzing public health policies [37–39]. It allows examining “the way that language functions in order to shape the public perceptions of health issues and the policy debates that surround them” [40]. In this study, CDA allowed us to critically examine and interpret the ways migrants’ SRHR were framed and addressed in Swedish SRHR-related policies. As stressed by Fairclough [33] CDA “is part of some form of systematic transdisciplinary analysis of relations between discourse and other elements of the social process.” In this sense, CDA allowed us to consider the wider social context when performing the analysis.

## Materials

The search for materials focused on three websites: the National Board of Health and Welfare’s website, the Government Offices of Sweden's website, and the Public Health Agency of Sweden's website. The search was conducted in English and Swedish. The search terms used were *sexual health, reproductive health, sexual and reproductive health, sexual and reproductive rights, sexual and reproductive health and rights, migrant health, newcomers’ health, contraception,*^3^*abortion*^*3*^*, and maternal health*^*3*^*.* This search was supplemented by examining the areas “gender equality”, “youth policy”, “public health and medical care”, “introduction of new arrivals”, and “migration and asylum” under the column “government’s policy” in the website the Government Offices of Sweden. In this website, it was possible to limit the search by the type of the document. Documents presenting country and regional strategy, legal document, statement, committee report, case list, government assignment, and agreements were included. We aimed to include all SRHR-related policy documents published starting from the mid-90 s until 2020. However, as the search relied on online websites, the ability to find documents published in the period between mid-90 s and late-90 s was very limited.

A total of 277 documents were saved after screening by title. We used the concept of SRHR to guide the screening step [29]. Throughout the search process, SRHR were conceptualized by referring to the Guttmacher-Lancet Commission’s definition presented above [29]. The search was supplemented by including the following search sites related to gender based violence, considered an important component of SRHR [29]: the National Center for Knowledge on men’s violence against women, the Swedish Gender Equality Agency, the County Administrative Board of Östergötland that has been in charge since 2005 of working on preventing and counteracting “honor-related violence and oppression” at the national level, and the website “hederförtryck.se” (honor oppression.se)- an information website launched by the County Administrative Board of Östergötland.

**Fig. 1:**
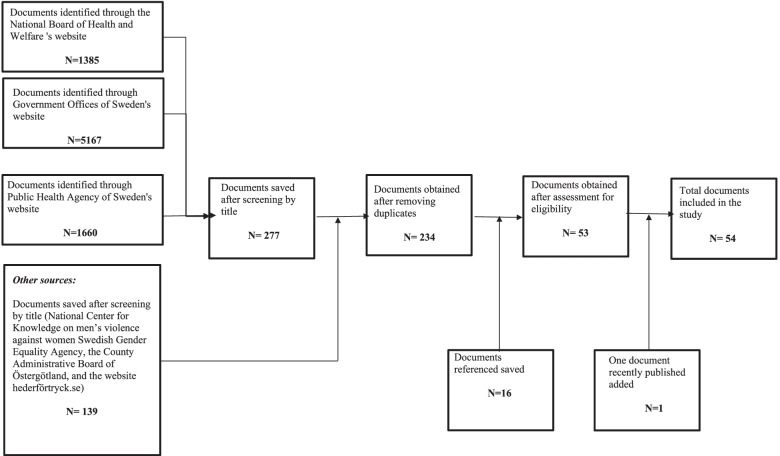
Diagram illustrating the data collection process

After removing the duplicates, 234 documents were obtained. Moreover, 16 documents referenced in the documents saved were added to the pool to be examined for inclusion. The first author examined these documents to determine whether they were relevant to the study or not. Documents were included if they 1) represent policy documents defined as i.e., national strategies, policies, action plans, policy assignments, government’s bills, and appointed committee’s reports and 2) address topics related to migrants’ and people with a foreign background’s SRHR. A total of 54 documents were included in the analysis. The following diagram illustrates the search process (Fig. 1):

### Analysis

The analysis in this paper focused on the analysis of discourses used to represent migrants as well as to frame and address their SRHR-related issues. Discourses were understood as a series of statements related to migrants with regard to SRHR. Within the CDA approach, diverse methods and approaches were proposed to analyze discourses [33, 41]. In this study, the analysis process was guided by Jäger’s approach [42] which facilitated examining the structures of different discourses within Swedish SRHR-related policies. According to this approach, the structure of a discourse can be identified using discourse strands, discourse fragments, and entanglements of discourse strands. Discourse strands refer to “thematically uniform discourse processes” [42], for example, a human right-based discourse strand. Discourse fragments refer to a text or a part of text addressing a specific theme. Multiple discourse fragments can be combined to form discourse strands. Entanglements illustrate how different discourse strands can intersect or crosscut in a text [42].

**Fig. 2:**
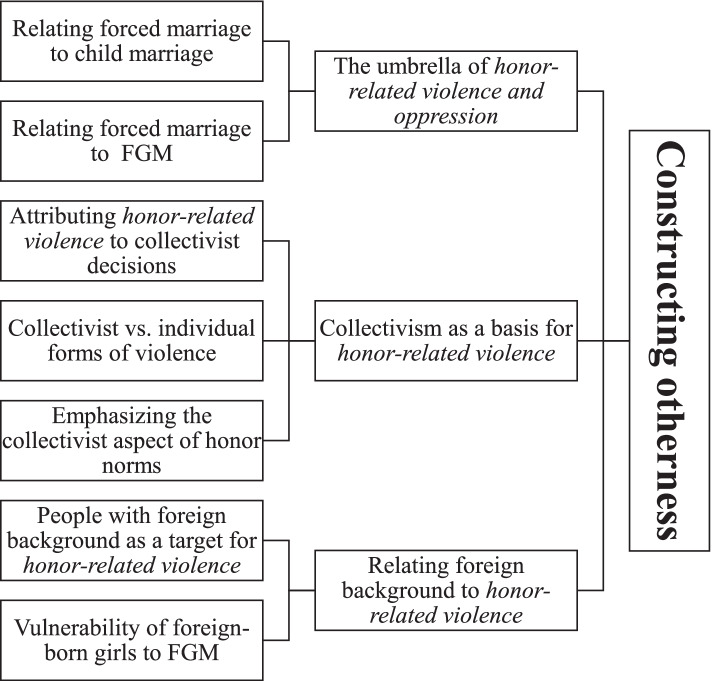
From codes to a discourse strand: an illustrative figure of the analysis process

First, selected documents were read, and sections related to the SRHR of migrants and people with a foreign background were identified. Second, selected documents were imported to the software NVivo 12 [43]; and identified sections were coded. The first documents were coded inductively [44]. The inductive codes were used to code the rest of the documents with adding new codes that emerged during this process. Third, codes were examined to look for patterns in the data. The analysis focused on the most important patterns that could reflect dominant discourses. An example of patterns identified is “collectivism as a basis for *honor-related violence*”. This pattern was associated with codes “attributing *honor-related violence* to collectivist decisions”, “collectivist vs. individual forms of violence”, and “emphasizing the collectivist aspect of honor norms”. Related patterns were grouped to form discourse strands (Fig. 2). Finally, the discourse strands were examined to identify potential entanglements.

## Results and discussion

The findings consisted of three discourse strands: 1) “Emphasizing vulnerability”, 2) “constructing otherness”, and 3) Prioritizing the structural level or the individual level?” In this section, discourse strands are presented and discussed. Two entanglements between different discourse strands are also described.

### Migrants in Swedish SRHR-related policies

In this section, we provide a description of the terminology used to refer to migrants in Swedish SRHR-related policies. Overall, policy documents examined in the analysis have often used the terms “foreign-born” and “people with a foreign background” to refer to migrants and to a larger group, including people with two parents born outside of Sweden. These terms seem to replace the term “immigrant” or “*invandrare*” that was questioned since the 90 s, especially when referring to people born in Sweden to foreign-born parents, often called “second generation immigrants” or “immigrant children” [45, 46]. In the documents analyzed, the term “immigrant” was only found in the policy documents published in the early- and mid- 2000s. A term that stands out in the analyzed policy documents is “persons or individuals with migration experiences” which has been used in the national SRHR strategy published in 2020. Although not clearly defined, this term seems to refer to a broader category of migrants that extends beyond those who have settled in Sweden. This term also seems to be employed in an attempt to capture different aspects of the migration process from departing from the home country to the establishment in a new country as outlined in the following quote:

“Migration processes, from departure to establishment in a new society, can involve or risk of ill health and sexual exposure. Some groups, of those who have migration experience, sometimes have poorer health than the rest of the population”. (Nationell strategi för sexuell och reproduktiv hälsa och rättigheter (SRHR), 2020).

Migrants are not viewed as a homogenous group in the dominant discourses embedded within Swedish SRHR-related policies. This was indicated by the use of different terms related to different categorizations of migrants that are not only based on the legal status, but also on gender, age, and length of stay in Sweden. Examples of these terms include undocumented migrants, asylum seekers, unaccompanied minors, and newcomers or newly arrived migrants.

### Emphasizing vulnerability

In relation to SRHR, vulnerability seems to be an important feature of migrants and people with a foreign background in Swedish SRHR-related policies. The vulnerability of some migrant groups is specially highlighted. For instance, migrant women and girls are considered as a special vulnerable group to what is called honor-related violence and oppression and to violence against women. Overall, foreign background is considered as a factor contributing to the vulnerability to violence against women as mentioned in the “National strategy to prevent and combat men’s violence against women”:

“Circumstances linked to foreign background and age also affect exposure to violence”. (Skr. 2016/17:10)

As shown in the previous quote, not only gender, but also age was considered as a marker of vulnerability. Young newcomers and migrant youth are portrayed as a vulnerable group in relation to SRHR in general, whereas unaccompanied minors and underaged asylum seekers are considered a vulnerable group to sexual exploitation, sexual abuse, and human trafficking. Nevertheless, in the national Action plan against prostitution and human trafficking, unaccompanied minors were also considered as “a risk group” as mentioned below:

“Unaccompanied children have been identified as a risk group for prostitution and human trafficking”. (Handlingsplan mot prostitution och människohandel, 2018)

Interestingly, in relation to HIV and STIs, there was a clear shift from depicting people with a foreign background, especially newcomers and asylum seekers, as a risk group in the HIV strategy published in 2005 to emphasizing migrants as vulnerable group in the revised strategy published in 2017. This shift was associated with narrowing the target group from newcomers and asylum seekers in general to migrants and newcomers coming from specific regions defined as HIV endemic countries. It was also associated with changing focus from risk identification to prioritizing prevention and reducing vulnerability. Risk identification was driven not only by the concern of protecting the migrants, but also by the concern of protecting the society from potential infection risks as illustrated by the following excerpt:

“According to the Government's assessment, it is a matter of course that people with a foreign background who are judged to be at risk should be offered a health examination which includes HIV testing and counseling. Such an offer is important both for the individual and for society's infection control”. (Prop. 2005/06:60).

In the national SRHR strategy published in 2020, the term “vulnerable groups” was replaced by the expression “groups whose health and rights need to be strengthened”. These groups, which are not restricted to migrants, are considered disadvantaged in terms of health and rights as explained in the following excerpt:

“However, there are groups in society whose sexual and reproductive health is worse than that of the rest of the population and whose rights are more often neglected. These groups therefore need to be strengthened. These are, for example, people with inadequate socio-economic conditions, people with migration experience, people with disabilities, LGBTQI people and young people”. (Nationell strategi för sexuell och reproduktiv hälsa och rättigheter (SRHR), 2020).

It is not clear whether this change reflects a problematization of the concept of vulnerability or an attempt to clarify this concept with regards to SRHR. The concept of vulnerability has been exhaustively discussed in the literature as it carries different and often contradicting connotations and meanings. For instance, Leinonen & Pirjatanniemi [46] argue that within the contemporary human rights discourse, the concept of vulnerability is employed to “refer to individuals and groups whose rights are perceived to be at a particular risk, such as refugees, undocumented migrants, or migrant children”. This conceptualization of vulnerability is highlighted in the Swedish discourses on migrant’s SRHR, where the rights of migrants or some groups of migrants, such as migrant women or unaccompanied minors, are viewed to be at a particular risk. This conceptualization also seems to be rooted in the human rights-based approach embraced by Swedish SRHR-related policies.

However, the strong emphasis on migrants’ vulnerability in Swedish SRHR-related policies might have negative implications. For instance, the concept of vulnerability is viewed as a paternalistic label that can reinforce stigmatization and social exclusion, and can convey an image of passivity, dependency, and disempowerment [47, 48]. These criticisms can be particularly relevant to how the analyzed SRHR-related policy documents often stressed migrant women’s vulnerability to gender-based violence and what is defined as honor-related violence. While this emphasis might be driven by a concern to ensure migrants’ SRHR, it might contribute to constructing migrant women as passive lacking agency. The tendency to deploy the discourse of victimhood when depicting migrant women in public policies was highlighted in previous literature. For instance Shrover [49] and Roggeband and Verloo [50] showed how Deutsch policies used gender equality and women’s rights discourse to emphasize the image of migrant women as passive and victims of a patriarchal Muslim culture.

The concept of vulnerability might also operate as a social control mechanism. It can set the ground for interfering’s in people’s lives and singling out some groups [48, 51, 52]. This can be particularly relevant for migrants and newcomers migrating from what is identified as HIV endemic countries where the label of “vulnerable group” can be used to justify singling out and practicing some forms of social control over this group.

The vulnerability of different migrant groups is explained by multiple factors operating at different levels. For instance, in the policy document “Domestic violence-A public health issue”, individual-level factors such as language difficulties, lack of knowledge about the Swedish system and laws, social isolation, and restricted social network were presented as factors contributing to the vulnerability to violence against women among women with a foreign background. This policy document also pointed to the role of structural level factors, including discrimination, segregation, and lack of economic resources, and underlined how migrant women’s vulnerability might be exacerbated by the migration’s regulations as illustrated by the following quote:

“(…) in Sweden the women who are exposed to violence but do not dare to leave the man before the two-year period is over, as they then risk being deported”. (SOU 2014, 49)

The regulation related to the two-year period, often called the two-year rule, refers to the requirement for a migrant to live with a partner for at least two years before obtaining a permanent residence permit [16]. This rule has been criticized for creating a situation of dependency where migrant women might be obliged to stay in a violent relationship until obtaining a permanent resident permit [53].

Constructing migrants and people with a foreign background as a vulnerable group has implications. According to Brown [48], “the concept of vulnerability informs how we manage and classify people, justify state intervention in citizens’ lives, allocate resources in society and define our social obligations”. Accordingly, the concept of vulnerability may define the social obligations towards migrants in the SRHR arena and might justify migrants’ entitlements to specific resources. In Swedish SRHR-related policies, the state is perceived as the main responsible for protecting the vulnerable groups’ rights, which suggests that the concept of vulnerability might be used positively to hold the state accountable for advancing migrants’ SRHR.

Although the dominant discourses embedded in Swedish SRHR-related policies stresses the state’s obligation in protecting the rights of different migrant groups, choosing to use of the term “vulnerable” instead of other terms such as “underserved”, can emphasize the idea of individual or personal responsibility. The concept of vulnerability has been criticized for shifting the attention from structural and systemic issues towards focusing attention on the individual [48, 54–56]. These implications structural level vs. individual level are discussed in the third discourse strand.

### Constructing otherness

Within Swedish SRHR-related policies, the discourse of otherness and othering appears mainly when addressing highly culturalized issues such as FGM, child marriage, forced marriage, and what is called honor-related violence. In this study, othering is understood as “discursive processes by which powerful groups, who may or may not make up a numerical majority, define subordinate groups into existence in a reductionist way which ascribe problematic and/or inferior characteristics to these subordinate groups” [57]. Othering involves distancing from and stigmatizing the Other to secure one’s own identity [58], as well as constructing the self and the Other in a mutual and unequal opposition [59].

In SRHR-related policies, issues like FGM, child marriage, forced marriage are conceptualized as interrelated and are placed under the umbrella of what is defined as honor-related violence and oppression. The concept of honor-related violence was introduced in the Swedish discourse in the late 90 s after three cases of murders qualified as honor killings [60–62]. Since then, the honor-related violence has been a topic of intensified debates in the Swedish context.

The issues of what is called honor-related violence and oppression and its different features are mainly discussed within the culturalist framework. They are viewed to be related to first and foremost to collectivist thinking and norms as exemplified by the quotes below:

“The basis of honor norms is the subordination of the individual to the collective consisting of the family. The concept of family does not only include not only the immediate family but also other relatives”. (SOU 2014: 49).

Within the discourse of otherness, the issue of what is called honor-related violence and oppression is portrayed as a serious social problem and a special form of violence or oppression that differs from other forms of gender-based violence or violence in close relationships. The following excerpt shows how honor-related violence and oppression was presented as a “*collective-based violence*” that can be “*socially accepted*” by some groups contrarily to other individual-based forms of violence:

“Violence and oppression [Honor-related violence and oppression] can thus be described as collective-based (several perpetrators) in contrast to other forms violence against close relatives that are individual-based (one perpetrator). It can also be described as rooted in a wider circle (it is socially accepted in the context in which it is practiced and supported by for example the family) as opposed to other forms of violence against close relatives that are condemned (it is disapproved of by the perpetrator and the victim's relatives). Finally, honor-related violence and oppression is often planned in contrast to other forms of violence against relatives”. (SOU 2018:69).

This collectivist thinking and norms are related to the culture of the “Other” that are thought to be “problematic” and different from the Swedish values and norms. The “Other” in this context refers mainly to foreign-born people and people with a foreign background as illustrated, for example, by the way newcomers and asylum seekers are depicted in the “National strategy to prevent and combat men’s violence against women”:

“Many newcomers and asylum seekers have grown up in societies where the protection of women's and girls' and LGBTQ people's physical integrity, sexual self-determination and position is in general significantly weaker than in Sweden”. (Skr. 2016/17:10).

To emphasize the particularity of this issue, a broad range of terms were deployed including “*honor norms*”, “*honor thinking*”, and “*people living in honor context*” reflecting what Carbin [61] called “a discursive explosion” in the debates and discussions surrounding what is defined as honor-related violence. The Swedish language was, therefore, “enriched” by new words related to what is considered as honor-related violence [61]. These terms/concepts “constituted a line of demarcation separating the Swede from its others” as stated by Alinia [63], and were primarily related to controlling women’s sexuality and behaviors as mentioned below:

“Control of girls' and women's sexuality is a central feature of honor contexts.” (S2018/03931/JÄM)

Within the discourse of otherness, the use of the dichotomy majority/ minority is recurrent as illustrated by the use of the terms “*ethnic and religious minorities*” vs. “*the majority population*” or the terms “*the majority culture*” vs. “*the minority culture*”.

In addition to challenging honor norms and providing support to women and girls, integration was presented as a key solution to tackle the root causes of what is conceptualized as honor related violence and oppression. This integration process starts with the social orientation course received by the newcomers, which explains the focus on incorporating individual rights including sexual and reproductive rights and issues related to violence as well as gender equality in this course as stated in the government’s “Action plan to prevent young people from being forced into marriage”:

“The Government believes that social orientation for newly arrived migrants should convey the importance of fundamental values ​​such as democracy and the equal value of all people. It should include knowledge and reflection on what it means to live in the Swedish society, gender equality and respect for young people's integrity. It should give clear information about the rights and obligations of the individual”. (S kr. 2009/10:229).

Following the same line of thought, segregation was related to increased risk of what is defined as honor related violence and working on reducing segregation is presented as the solution for this issue as stated below:

“Increased segregation can lead to strengthen honor norms in economically disadvantaged areas. Therefore, it is important to promote gender equality and challenge honor norms within the framework of initiatives for both women and men in the work with the Government's long-term reform program for reduced segregation 2017–2025”. (Skr. 2016/17:10).

While there was no clear shift over time in the dominant discourse of otherness, this discourse has been challenged by a less prevailing yet competing discourse that strives to move away from the reductionist approach in addressing issues related to what is defined as honor-related violence and oppression. Within this challenging discourse, there is more emphasis on the heterogeneity of people with a foreign background and foreign-born people. There is also an attempt to dissociate the issue conceptualized as honor-related violence and oppression from a particular culture and/or religion as exemplified in the following excerpt:

“Honor thinking can take different forms depending on cultural positions and religion but is not linked to any specific culture or religion. Honor thinking can also occur in non-religious contexts.” (Skr. 2007/08:39).

Within this discourse, adopting a norm critical perspective is also seen as a way to address issues related to what is defined as honor-related violence and oppression. Additionally, adopting a more holistic approach in addressing these issues is prioritized as stated in the “National strategy to prevent and combat violence against women and honor-related violence and oppression”:

“The initiatives to prevent violence with a holistic perspective must be implemented to a greater extent to reach relevant target groups”. (SOU 2015:55)

The discourse on migrant vulnerability and the discourse of otherness converge when addressing issues linked to what is defined as honor-related violence, leading to the emergence of what can be interpreted as the “vulnerable other”. The “vulnerable other” is people with a foreign background who are constructed as particularly vulnerable to the issue of what is called honor-related violence and oppression as mentioned below:

“The fact many of those who are exposed to crimes with honor motives have a foreign background must not affect the public's approach to such crime”. (SOU 2018:69)

This seems to reflect the representation of people with a foreign background in the public discourse that was advanced by Khayati [64] who argues that public discourses on migration in Sweden have reduced people with a foreign background to a culturally, ethnically, and religiously "deviant" social category, with limited opportunities for exercising agency. Previous literature has shown how SRHR issues, mainly violence against women and what is called honor-related violence and oppression have been subjected to othering in the Swedish context [63, 65]. Karlsson et al. [65] argue that there is a tendency for othering intimate partner violence in the Swedish news media when the perpetrator in non-Swedish by “associating it with structural patterns located in non-Swedish rather than Swedish contexts”. Similar findings were highlighted in a policy analysis examining EU policy texts addressing violence against women, where the culturalization of this form of violence led to the emergence of the dichotomy “non-violent Europeans” vs. “violent others” [66].

Constructing people with a foreign background as particularly vulnerable to what is defined as honor-related violence and oppression can set the ground for singling out them in the healthcare setting and in the social services as exemplified in the following excerpt:

“At the first health visit with a girl who comes from one of the countries where genital mutilation occurs, you can ask if the girl knows anything about genital mutilation or if she herself has any experience of it”. (Kvinnlig könsstympning – ett stöd för hälso- och sjukvårdens arbete).

Although this measure is intended to address FGM, the risk of stigmatizing some groups of patients needs to be carefully considered. For instance, a study conducted in the U.K exploring the impact of FGM-safeguarding measures in healthcare settings on people with a British-Somali heritage showed that participants viewed the routine and often-repeated questions on FGM as invasive and insensitive [67]. The FGM-safeguarding measures were also viewed as relying and reinforcing stereotypes surrounding the Somali community [67].

### Prioritizing the structural level or the individual level?

The discourses framing migrants’ issues with regards to SRHR oscillate between two competing discourses. The first one tends to prioritize the structural level, as mainly reflected in adopting the human rights-based discourse, whereas the second tends to frame migrants’ SRHR issues and needs as individual issues requiring individual-level solutions.

Within the human rights-discourse, the state is considered “a duty bearer” accountable for the fulfillments of individuals’ and communities’ rights through its policies, strategies and programs, whereas individuals and communities are considered “right-holders” who can claim these rights [68]. This approach suggests that SRHR issues can be conceptualized as structural issues requiring structural change through working on factors located beyond the individual at, for example, the legal and policy spheres [29].

The human rights-based discourse is a dominant discourse in Swedish SRHR-related policies. It represents the main framework for framing these policies. This tendency is clearly reflected in the frequent use of international human rights-related conventions (e.g., the Council of Europe Convention on preventing and combating violence against women and domestic violence “Istanbul Convention”, the Council of Europe Convention on Action against Trafficking in Human Beings, and the United Nations Convention on the Rights of the Child) as a starting point for Swedish SRHR-related policies in Sweden. These conventions are often presented in the section “*starting point*” of these policy documents.

In the policy documents analyzed, the human rights-based approach represents the main foundation for addressing migrant SRHR. For instance, issues such as child marriage, forced marriage, FGM, honor-related violence, and human trafficking are conceptualized as human rights violations as outlined, for example, in the following excerpt:

“Human trafficking is a serious violation of the individual's human value and right to decide over his or her life and body”. (Skr. 2007/08:167)

This discourse stressed that the response to migrants’ SRHR issues should be grounded in the human rights-based approach as exemplified in the following excerpt:

“In our knowledge base, it is clear that forced marriage and child marriage often have a link to honor-related violence and oppression. This knowledge is important for how the problems should be met. However, what is more important, completely apart from culture, religion, traditions and values, is to be based on the human rights of each individual; rights that have been ensured in several international conventions and recommendations—such as the UN Convention on the Rights of the Child, the UN Convention on the elimination of all forms of discrimination against women and the Council of Europe Convention on Human Rights and Fundamental Rights the freedoms”. (Prop 2013/2014:208).

The human rights-based approach has been also deployed to advance migrants’ access to SRH services as illustrated by the policy document “Health care for people staying in Sweden without a permit” published in 2013. In this document, the discourse on how to ensure undocumented migrants’ access to maternal healthcare, contraception, and abortion services was clearly driven by Sweden’s commitments to international conventions stressing the right to health and healthcare.

This tendency has been reinforced over time, which was reflected in the discourse employed in the new SRHR strategy published in 2020 where there was a strong emphasis on the human rights-based approach, presented as one of the foundations for achieving the strategy goals:

“The work to achieve the goals of the strategy must be permeated by human rights and more specifically by the rights linked to sexual and reproductive health. The right perspective is based on the principles of non-discrimination, participation, openness and transparency, as well as taking and demanding responsibility”. (Nationell strategi för sexuell och reproduktiv hälsa och rättigheter (SRHR), 2020).

The strategy has also defined “creating structural conditions for SRHR” as one of the measures to achieve the strategy goals. These “structural conditions” include developing local and regional plan actions to advance SRHR as well as ensuring a systematic and multidisciplinary work on issues SRHR. However, it is not clear how these measures can be operationalized with regards to migrants’ SRHR.

Although migrants’ SRHR issues are often framed within the human rights-based approach, the responses to these issues do not always reflect a thorough consideration of the structural factors. For example, as mentioned above, unaccompanied minors are depicted in Swedish SRHR-related policies as particularly vulnerable to human trafficking and prostitution. However, the discourse on how to address this issue omits the role of the migration process and the migration policies in constructing and exacerbating the vulnerability of this group. This was reflected in the measures suggested to address this issue that place a strong emphasis on increasing knowledge and competence in meeting the needs of this group without pointing clearly to the role of migration policies in this issue. As outlined by Ouis [16], young migrants’ and unaccompanied minors’ vulnerability might be the result of migration legislations restricting the opportunities to obtain a valid residence permit and leading to the emergence of what is called survival sex. Survival sex is a type of sex for compensation where the compensation consists of providing basic needs such as food and housing [16]. Failure to recognize how migration legislations and policies might have contributed to the vulnerability of unaccompanied minors and young migrants to human trafficking and prostitution might perpetuate this problem.

The tendency to disregard structural factors is also found in the discourse on HIV/AIDS where the notion of risk is still persistent in framing issues related to migration and HIV prevention. The risk-view to the prevention of HIV is rooted in the individualistic framework where individuals are considered “neoliberal rational agents” [69] who are able to change their “risky behaviors” when receiving information about HIV transmission and prevention and when having access to HIV-prevention tools such as condoms and sterile needles [69, 70]. This view is illustrated by the following excerpt from the revised HIV/AIDS strategy where the priority is given to providing information and knowledge when addressing migrants’ groups needs in relation to HIV prevention:

*“Collaboration between the public sector and non-profit organizations in those arenas who can reach these groups with target group-adapted information and knowledge about HIV infection and other sexually transmitted or blood-borne infections is important”.*

This individualistic framework also seems to shape the main response to the disadvantage of migrant youth and young newcomers in terms of SRHR as the focus has been on providing information and addressing the lack of knowledge. For instance, a multilingual online platform called “Youmo.se” was created to target young migrants and newcomers in matters related to SRHR. This platform provides information on, among others, health, human rights, SRHR, and gender equality. While the access to information and knowledge is important, this approach might fail to meet migrants’ SRHR needs by obscuring other salient socio-economic and migration related factors. Moreover, without linking access to information to other structural factors, this approach might reflect a model of governance favoring the discourse on “personal responsibility” [71], where migrants can be seen as fully responsible of their SRHR outcomes.

While migrant SRHR issues are occasionally conceptualized as structural issues, it is worth acknowledging that migrants might benefit from Swedish SRHR policies targeting the general population such as free contraceptive counselling and free HIV and STIs testing [72].

As explained above, the dominant discourses faming migrants’ SRHR situated migrants’ issues either at the individual level or at the structural level. Nevertheless, the dichotomy structural level vs. individual level might not capture all the conceptualizations of migrant issues, namely with regards to communication. The conceptualizations of communication issues with regards to migrants’ SRHR can reflect an interplay between different levels including not only the individual level and the structural level but also the interpersonal level. Language is an important feature of these communication issues. It is often depicted as “language difficulties” or “language barriers”. The discourse on how to address these barriers prioritizes increasing the accessibility of interpretation services and the translated materials for migrants as illustrated by the following excerpt:

“Equivalent information should be available in both easy Swedish and the most common mother tongues of newly arrived and asylum-seeking children and young people.” (S2016/02759/JÄM).

Communication issues were also conceptualized as intercultural communication issues where concepts, such as “cross-cultural competency” and “transcultural competence” or “interculturality,” were sometimes brought up when discussing working with migrants and people with a foreign background in issues related to SRHR. These concepts were not clearly in Swedish SRHR-related policies which might leave the door open for essentialist interpretations.

### Methodological discussion

The study has several limitations. First, the analysis was performed by the first author for whom Swedish is not the first language. Second, the documents were collected from online websites. This has limited the ability to collect policy documents published between the mid-90 s and late-90 s. However, to the best of our knowledge the main SRHR-related policies and strategies starting from early 2000s were included in the analysis. Third, although the search terms included terms like “contraception”, “abortion”, and “maternal health”, these areas were not highlighted in the findings. A potential explanation is that policies related to contraception, abortion, and maternal health did not address topics specific to migrants’ and people with a foreign background’s SRHR and were therefore not included in the pool of policy analysis to be analyzed. This might indicate that migrants were particularly visible when discussing some specific SRHR areas such as HIV/AIDS and what is called honor related violence and oppression.

Finally, in this paper, we presented an interpretation of the SRHR-related policy documents. We are aware that these texts can be interpreted differently, as in line with the CDA methodology “different understandings of the text result from different combinations of the properties of the text and the properties (social positioning, knowledges, values, etc.) of the interpreter.” [73].

## Conclusions

Our analysis has shown that the discourses on migrants’ SRHR emphasized the concept of vulnerability when portraying migrants and addressing issues related to their SRHR. The focus was on individual vulnerability without paying attention to the structural causes of this vulnerability, namely migration processes and legislations. This study calls for a thorough reflection on the use of the concept of vulnerability when developing policies related to migrants’ SRHR. A critical analysis of this concept can help advance migrants’ SRHR without bearing the negative consequences of stigmatizing or disempowering this group.

Alongside the discourse of vulnerability, the discourse of otherness appeared when discussing migrants’ SRHR, more specifically in relation to what is defined as honor-related violence and oppression. The discourse of otherness is in contradiction with the objective of the new Swedish SRHR strategy i.e., to achieve good and equal SRH in the entire population [31] and the global efforts to advance migrant health [2].

Finally, we found that the conceptualizations of migrants’ SRHR issues oscillated between prioritizing the structural level and emphasizing the individual level. Previous research has highlighted the important role of structural factors, such as migration policies and regulations, on migrant SRHR [13–15, 74] and migrant health in general [75]. For example, a systematic review and metanalysis has shown that restrictive entry and integration policies in high-income countries are associated with poor migrant health outcomes [75]. Nevertheless, these structural issues were sometimes occulted in Swedish SRHR-related policies when addressing migrants’ SRHR, such as HIV/AIDS and prostitution and human trafficking, which is in contradiction with the human rights-based approach presented as the main framework for these policies. This study points for the need to assess the effects of the structural-level factors on migrant SRHR in Sweden. Generating such context-specific evidence might help inform future SRHR-related policies.

## Supplementary Information

**Additional file 1: Table 1. **List of documents included in the analysis.

## Data Availability

The list of the policy documents used in the study is available in Supplementary file 1. All documents are available online.

## Abbreviations

AIDS: Acquired immunodeficiency syndrome
CDA: Critical discourse analysis
EU: European Union
FGM: Female genital mutilation
ICPD: International Conference on Population and Development
HIV: Human Immunodeficiency Virus
LGBTQ: Lesbian, Gay, Transgender and Queer/questioning
OECD: Organization for Economic Cooperation and Development
SRH: Sexual and reproductive health
SRHR: Sexual and reproductive health and rights
STIs: Sexually transmitted infections

## Footnotes

1 The term migrant is usually used to refer to foreign-born people in these studies.

2 It was not possible to distinguish the migrant groups from people born in Sweden to two foreign-born parents in the analyzed policy documents that used the term people with foreign background.

3 These search terms were added when we noticed that policy documents related to contraception, abortion, or maternal health were lacking in the pool of materials to be examined for inclusion. We decided to actively search for these policies by adding these three terms.
