# Supplementary material for: Migrants in Swedish sexual and reproductive health and rights related policies: a critical discourse analysis
Source: Int J Equity Health. 2022 Sep 5;21:125. doi: 10.1186/s12939-022-01727-z (PMC9446749; doi:10.1186/s12939-022-01727-z)
Supplement: Supplementary file 1 — Additional file 1: Table 1. List of documents included in the analysis. [file 12939_2022_1727_MOESM1_ESM.docx]

**Supplementary file 1:**

**Table 1: List of documents included in the analysis**

|  | **Document** | **Year of publication** |
| --- | --- | --- |
| 1 | Nationell handlingsplan mot våld i nära relationer SOU 2002:71 | 2002 |
| 2 | Utländska kvinnors rätt att få abort i Sverige Dir. 2003:134 | 2003 |
| 3 | Slag i luften. En utredning om myndigheter, mansvåld och makt SOU 2004:121 | 2004 |
| 4 | Makt att forma samhället och sitt eget liv – jämställdhetspolitiken mot nya mål SOU 2005:66 DEL 2 | 2005 |
| 5 | Nationell strategi mot hiv/aids och vissa andra smittsamma sjukdomar Prop. 2005/06:60 | 2005 |
| 6 | Abort i Sverige SOU 2005:90 | 2005 |
| 7 | Abort för utländska kvinnor och förebyggande av oönskade graviditeter Prop. 2006/07:124 | 2006 |
| 8 | Handlingsplan för att bekämpa mäns våld mot kvinnor, hedersrelaterat våld och förtryck samt våld i samkönade relationer Skr. 2007/08:39 | 2007 |
| 9 | Handlingsplan mot prostitution och människohandel för sexuella ändamål  Regeringens skrivelse Skr. 2007/08:167 | 2007 |
| 10 | Människohandel och barnäktenskap - ett förstärkt straffrättsligt skydd SOU 2008:41 | 2008 |
| 11 | Handlingsplan för att förebygga och förhindra att unga blir gifta mot sin vilja Skr. 2009/10:229 | 2009 |
| 12 | Nationell handlingsplan för klamydiaprevention – Med fokus på ungdomar och unga vuxna 2009–2014 | 2009 |
| 13 | Framtidens folkhälsa– allas ansvar En kortversion av Folkhälsopolitisk rapport 2010 | 2011 |
| 14 | Kvinnor som utsätts för våld efter att ha beviljats uppehållstillstånd i Sverige på grund av anknytning Dir. 2011:44 | 2011 |
| 15 | Stärkt skydd mot tvångsäktenskap och barnäktenskap SOU 2012:35 | 2012 |
| 16 | Hälso- och sjukvård till personer som vistas i Sverige utan tillstånd Ds 2012:36 | 2012 |
| 17 | Regeringens proposition 2013/14:191 Med fokus på unga –en politik för goda levnadsvillkor, makt och inflytande Prop. 2013/14:191 | 2013 |
| 18 | Ensam och utsatt Utbildningsmaterial om våld mot kvinnor med utländsk bakgrund | 2014 |
| 19 | Greater protection against forced marriage and child marriage | 2014 |
| 20 | Stärkt skydd mot tvångsäktenskap och barnäktenskap samt tillträde till Europarådets konvention om våld mot kvinnor Prop.2013/14:208 | 2014 |
| 21 | Våld i nära relationer – en folkhälsofråga Förslag för ett effektivare arbete SOU 2014:49 | 2014 |
| 22 | En förbättrad förlossningsvård och insatser för kvinnors hälsa Överenskommelse mellan staten och Sveriges Kommuner och Landsting 2015 och 2016 | 2015 |
| 23 | Mål och myndighet En effektiv styrning av jämställdhetspolitiken  SOU 2015:86 | 2015 |
| 24 | Nationell strategi mot mäns våld mot kvinnor och hedersrelaterat våld och förtryck  SOU 2015:55 | 2015 |
| 25 | Handlingsplan 2016–2018 till skydd för barn mot människohandel, exploatering och sexuella övergrepp | 2016 |
| 26 | Det handlar om jämlik hälsa Utgångspunkter för Kommissionens vidare arbete SOU 2016:55 | 2016 |
| 27 | Kvinnlig könsstympning – ett stöd för hälso- och sjukvårdens arbete | 2016 |
| 28 | Uppdrag om informationsinsatser rörande hälsa och jämställdhet för nyanlända och asylsökande barn och unga S2016/02759/JÄM | 2016 |
| 29 | En nationell strategi för att förebygga och bekämpa mäns våld mot kvinnor Diarienummer: Utdrag (kap 5) ur Skr. 2016/17:10 | 2017 |
| 30 | Folkhälsomyndighetens remissyttrande över slutbetänkande av Kommissionen för jämlik hälsa: Nästa steg på vägen mot en mer jämlik hälsa (SOU 2017:47) | 2017 |
| 31 | God och jämlik hälsa – en utvecklad folkhälsopolitik Prop.2017/18:249 | 2017 |
| 32 | Jämställdhetsmyndighetens remissvar på delbetänkandet utvidgat hinder mot erkännande av utländska barnäktenskap SOU 2017:96 | 2017 |
| 33 | Makt, mål och myndighet– feministisk politik för en jämställd framtid Regeringens skrivelse Skr. 2016/17:10 | 2017 |
| 34 | Nationell strategi mot hiv/aids och vissa andra smittsamma sjukdomar | 2017 |
| 35 | Nästa steg på vägen mot en mer jämlik hälsa SOU 2017:47 | 2017 |
| 36 | Regeringens arbete för att stärka socialtjänstens och hälso- och sjukvårdens insatser för ensamkommande barn och unga | 2017 |
| 37 | Regeringens proposition 2017/18:288 Förbud mot erkännande av utländska barnäktenskap Prop. 2017/18:288 | 2017 |
| 38 | Tilläggsdirektiv till Utredningen om starkare skydd mot barnäktenskap, tvångsäktenskap och brott med hedersmotiv Dir.2017:78 | 2017 |
| 39 | Underlag till handlingsplan mot könsstympning av flickor och kvinnor | 2017 |
| 40 | Utvidgat hinder mot erkännande av utländska barnäktenskap SOU 2017:96 | 2017 |
| 41 | Handlingsplan mot könsstympning av flickor och kvinnor S2018/03931/JÄM | 2018 |
| 42 | Handlingsplan mot prostitution och människohandel | 2018 |
| 43 | Länsstyrelsens återrapportering av uppdrag om översyn av samhällsorientering för nyanlända Dnr 851-1645-2018 | 2018 |
| 44 | Uppdrag om informationsinsatser rörande hälsa och jämställdhet för nyanlända och asylsökande barn och unga | 2018 |
| 45 | Uppdrag angående förstärkta informationsinsatser rörande hälsa, sexualitet och jämställdhet riktade till unga nyanlända och unga asylsökande S2016/02759/JÄM | 2018 |
| 46 | Uppdrag angående effektiva arbetssätt och metoder för informationsspridning gällande könsstympning av flickor och kvinnor S2018/03926/JÄM | 2018 |
| 47 | Ökat skydd mot hedersrelaterad brottslighet SOU 2018:69 | 2018 |
| 48 | Förebyggande arbete mot könsstympning av flickor och kvinnor Rapport 2019:9 | 2019 |
| 49 | Straffansvar för hedersrelaterat våld och förtryck Dir. 2019:43 | 2019 |
| 50 | Uppdrag att stärka arbetet mot att barn och unga respektive vuxna utnyttjas i prostitution och människohandel samt kartlägga omfattningen av prostitution och människohandel | 2019 |
| 51 | Regeringens proposition 2019/20:131 Ökat skydd mot hedersrelaterad brottslighet | 2020 |
| 52 | Skärpta regler om utländska månggiften SOU 2020:2 | 2020 |
| 53 | Nationell strategi för sexuell och reproduktiv hälsa och rättigheter (SRHR) | 2020 |
| 54 | Ökad tillgänglighet och jämlikhet i förlossningsvården och förstärkta insatser för kvinnors hälsa Överenskommelse mellan staten och Sveriges Kommuner och Landsting 2017- 2019 | ---- |
